# Improving local health through community health workers in Cambodia: challenges and solutions

**DOI:** 10.1186/s12960-017-0262-8

**Published:** 2018-01-06

**Authors:** Kim Ozano, Padam Simkhada, Khem Thann, Rose Khatri

**Affiliations:** 1 Public Health Institute, Liverpool John Moores University, Henry Cotton Campus, 15-21 Webster Street, Liverpool, L3 2ET UK; 2 Louvain Cooperation, #17, Street 282, BKK I, Chamkarmorn, P.O. Box 1e12, Phnom Penh, Cambodia; 3 Public Health Institute, Liverpool John Moores University, Henry Cotton Building, 15-21 Webster Street, Liverpool, L3 2ET UK; 4 Public Health Institute, Liverpool John Moores University, Henry Cotton Building, 15-21 Webster Street, Liverpool, L3 2ET UK

**Keywords:** Community health workers, Cambodia, Health promotion, Health systems, Qualitative research

## Abstract

**Background:**

Volunteer community health workers (CHWs) are an important link between the public health system and the community. The ‘Community Participation Policy for Health’ in Cambodia identifies CHWs as key to local health promotion and as a critical link between district health centres and the community. However, research on the challenges CHWs face and identifying what is required to optimise their performance is limited in the Cambodian context. This research explores the views of CHWs in rural Cambodia, on the challenges they face when implementing health initiatives.

**Methods:**

Qualitative methodology was used to capture the experiences of CHWs in Kratie and Mondulkiri provinces. Two participatory focus groups with CHWs in Mondulkiri and ten semi-structured interviews in Kratie were conducted. Results from both studies were used to identify common themes. Participants were CHWs, male and female, from rural Khmer and Muslim communities and linked with seven different district health centres.

**Results:**

Findings identify that CHWs regularly deliver health promotion to communities. However, systemic, personal and community engagement challenges hinder their ability to function effectively. These include minimal leadership and support from local government, irregular training which focuses on verticalised health programmes, inadequate resources, a lack of professional identity and challenges to achieving behaviour change of community members. In addition, the CHW programme is delivered in a fragmented way that is largely influenced by external aid objectives. When consulted, however, CHWs demonstrate their ability to develop realistic practical solutions to challenges and barriers.

**Conclusions:**

The fragmented delivery of the CHW programme in Cambodia means that government ownership is minimal. This, coupled with the lack of defined core training programme or adequate resources, prevents CHWs from reaching their potential. CHWs have positive and realistic ideas on how to improve their role and, subsequently, the health of community members. CHWs presented with the opportunity to share learning and develop ideas in a supportive environment would benefit health initiatives.

## Background

The ‘Health for All’ principles agreed in the Alma-Ata declaration (1978) set out a global determination to tackle the wider social, economic, political and environmental determinants of health through a community-based, participatory comprehensive primary health care (PHC) system [1]. In the absence of sufficiently trained health professionals, many low- and middle-income countries (LMICs) developed community health worker (CHW) programmes [2]. CHWs continue to be the frontline health workers in many LMICs, particularly for under-served rural populations [3–5]. CHW tasks vary from country to country but generally include management of uncomplicated childhood illnesses, advice on maternal and new-born care and prevention and treatment of non-communicable and communicable diseases such as malaria, tuberculosis (TB) and HIV/AIDS [3, 6–11]. The importance of identifying successful CHW programmes in LMICs is increasing in light of the Sustainable Development Goals (SDG) and particularly the aspiration to achieve universal health coverage [10, 12, 13]. However, research to understand the challenges faced by this workforce and their views on how to optimise performance is limited [3, 14–16].

The CHW programme in Cambodia is part of the wider national ‘Community Participation Policy for Health’ [17, 18]. The policy states that CHWs should be literate, live in the communities they serve and be elected by community members. Each CHW serves between 10 and 50 households depending on the community need. The CHWs are not a homogeneous group and vary widely in age, gender, experience, social status and political affiliation. Programme implementation should be financed and supported by Provincial Health Departments (PHD) and the administrative Operational District (OD) in terms of structure and management. Each CHW is associated with a health centre where they should receive training and supervision and in some cases resources to support their role [18]. Alongside this system, international and national non-government organisations (NGOs) build CHW capacity and finance activities to support their health-based initiatives [18].

This paper presents findings from two studies with CHWs in two eastern provinces of Cambodia and identifies how contextual factors influence the implementation of the CHW programme. It aims to highlight the scope of activity undertaken by CHWs in Cambodia and the variety of crosscutting skills needed to achieve these. The paper explores the perceptions, experiences, barriers and challenges faced by CHWs when implementing health initiatives in two districts. Their ability to identify practical realistic solutions to improve projects and even make links between projects is discussed. The implications of presenting CHWs with a supportive environment to develop ideas and improvements of health programmes in Cambodia adds to the body of research advocating for CHW voices to be heard from project planning to evaluation [19, 20].

### Cambodia

Cambodia’s history and development is complex with colonial legacies and more recent conflicts influencing societal relations. Between 1975 and 1979 under the Khmer Rouge Communist regime, Cambodia experienced one of the worst genocides in recent history with approximately a quarter of the population executed. Personal and political freedoms were outlawed, and intellectual cleansing resulted in the mass execution or exodus of the educated classes [21]. Following the war, Cambodia began to rediscover and reshape its identity under the auspices of the United Nations (UN) [22, 23]. The Ministry of Health was formed, and in 1994, the government set out a new determination to meet the needs of people living in remote areas who previously had no access to health services [24]. Although the development of Cambodia in health and other sectors has improved the lives of many, challenges still exist.

At the time of this article (2017), Cambodia ranks as a lower middle-income country with 41% of the population living close to, on or beneath the poverty line, measured at US$ 2 per day, the majority of which live rurally [25, 26]. Even with recent health improvements, Cambodia still has one of the highest infant and under-five child mortality rates in the Western Pacific region with 35 per 1000 children dying under the age of 5 [26, 27]. Maternal mortality rates remain high at 170 per 100 000 live births compared with the regional rate of 49 [27, 28]. Nevertheless, Cambodia is going through a demographic and health transition with falling fertility rates, increased life expectancy and an ageing population [29, 30]. There is also a dual burden of disease with communicable diseases like tuberculosis among the highest in the world [31] occurring alongside an increase in non-communicable diseases which now account for approximately 53% of deaths per year MoH and WHO; [29]. Health inequalities in rural areas, where 80% of the population live, are significant and a consequence of an unfair distribution of income, resources and other determinants of health, including clean water, nutritious food, adequate housing, public health services and adequately trained health personnel [25, 27, 32–36].

As part of health system development, in 2003 (revised in 2008), the *Community Participation Policy for Health* was created and implemented, highlighting a key role for CHWs [17, 37]. Table 1 reflects the potential variety and scope of skills and knowledge required to undertake the duties of a CHW in Cambodia. Although referred to as Village Health Support Group (VHSG) members in the policy, the generic term CHW is used here to allow the cross comparison of this role against the ambitions of community participation in the Alma Ata Declaration.

**Table 1:** Potential scope of work for CHWs adapted from the Community Participation Policy for Health

Health information systems: • Disease surveillance/monitoring and case reporting to the health centre • Keep a register of all children below 5 years of age in the village • Assist the health centre in collecting registration statistics including notification of pregnancies, births and deaths • Conduct verbal autopsies for deaths that occur in the village • Collect information on health and health-related problems in the community, inform and report to the health centre
Provision and follow up of information and essential services: • Facilitate the identification of the poor for fee exemption • Provide health education, promote improved health practices and distribute health IEC materials including family planning, antenatal care, clean delivery, post-natal care, breastfeeding, complementary feeding, safe water, hygiene and sanitation, malaria and dengue control, HIV/AIDS/STIs, tuberculosis, immunizations, non-communicable and chronic diseases, mental health, tobacco and alcohol and gender-based and family violence • Mobilise families and assist health centre staff during outreach activities and health campaigns • Assist in the mobilisation of resources for sustainability of health centres • Assist families with early identification of the danger signs for severe/serious illnesses • Promote and strengthen the health centre referral system and assist in logistics such as transportation
Provision and follow-up of essential diagnosis and treatment services: • Promote correct home care for illnesses • Provide community-based first aid and rehabilitation • Identify, refer and follow up children with acute malnutrition • Provide home-based care
In remote and difficult to access communities: • Provide early diagnosis and treatment for malaria • Diagnosis and treat acute respiratory infections with antibiotics in children
Provision of essential commodities: • Distribute micronutrient and food supplementation • Distribute mebendazol and oral re-hydration treatment with zinc • Distribute condoms and family planning supplies • Distribute long-lasting insecticide-treated mosquito bed nets and hammock nets

It is important to recognise that this is being asked of ‘lay’ volunteer health workers with limited education, some not beyond primary level. Also, CHWs are often close to the poverty line themselves, which places them at risk of reducing their income further when much of their time and resources is spent doing voluntary work [38, 39]. Furthermore, the socio-cultural context of Cambodia, including kinship, social hierarchical structures, religion, patron-client relations and collectivism [40–42], has an impact on the ability of communities to voice their concerns and participate in decisions that affect their health. CHWs have a role to advocate for health improvements on behalf of communities; however, the level of participation and influence that CHWs can have depends on international, national and local agencies engaging them in dialogue from policy making to implementation and evaluation.

Published literature of CHW activity and performance in Cambodia is limited. However, a small number of reports by NGOs and other published research have demonstrated that they achieve positive health outcomes when supported appropriately [43–46]. For example, a large-scale programme implemented with an international partner to improve child survival in Cambodia found that by providing 46 CHWs with training and regular supervision with supportive feedback, 2465 children were successfully treated for a number of ill health conditions including diarrhoea and pneumonia [44]. A study of eight rural health centres in Cambodia also found that higher levels of community engagement by CHWs in Cambodia resulted in better utilisation of health services, improved staff attitudes towards users and more efficient functioning of health centres [37].

Financial and managerial constraints hinder governmental support of the CHW programme which increases dependency on external donor support from some of the major international NGOs (INGOs) [47, 48]. This includes the provision of financial and technical support for capacity building and supervision, facilitating community organisation by linking health actors together such as health centre staff and CHWs with the community and providing management support to health centres and sub-national administrative teams [37, 49–51]. However, initiatives delivered by international partners are often short term with limited geographic coverage and resources [45, 50–52], which presents a risk to the sustainability and future of these programmes [50–52].

Consequently, many performance barriers are faced by health workers including fragmentation of service delivery and structure, inadequate financial remuneration and materials, lack of structured professional development opportunities and regular training, and poor supervision and management. This is further compounded by low community status and appreciation and negative patient/community member attitudes coupled with low levels of education leading to a lack of understanding of health messages [53–55]. This is consistent with research highlighting challenges experienced by CHWs globally which include high demands from external agencies, low literacy levels of community members and family/work commitments which impact on CHWs capacity to implement national policy objectives [14, 15, 56]. Furthermore, the CHW programme in Cambodia does not have an accreditation system, an initial training package or regular structured ongoing training and remuneration as recommended and found in other countries [5, 7, 57]. In order to address such challenges, a large-scale qualitative evaluation of health workers in Cambodia suggests that policy makers and partners should listen and apply the suggestions voiced by health workers in order to improve their working conditions and satisfaction which in turn could lead to improved health care [54].

## Methods

An interpretive, descriptive qualitative study design [58] was applied to gain insight into the perceptions, experiences, barriers and challenges faced by CHWs when implementing health initiatives in Kratie and Mondulkiri provinces. These provinces are mainly rural, experience significant socio-economic challenges and have higher maternal and child mortality rates in comparison to other provinces [27]. Poor infrastructure, including difficult roads and limited access to clean water and nutritious food, present additional challenges to CHWs [27]. Kratie and Mondulkiri provinces are also home to a large proportion of indigenous and Khmer Cham Muslim populations [59].

The study employed qualitative methods based on the interpretivist paradigm which considers the subjective experience of participant and their interpretation of social reality as critical [7].Qualitative methods included two focus groups with CHWs in Mondulkiri as part of an under-five malnutrition project and ten semi-structured interviews in Kratie as part of a doctoral study. Although these were separate studies, they fulfilled the same research question and objectives regarding the role and function of CHWs.

The research team consisted of one UK-based researcher and three Cambodian research assistants (RAs) who facilitated the qualitative research in the Khmer language. The RAs were trained in qualitative interviewing and focus group techniques over a 2-week period with opportunities to practice with fellow Khmer colleagues to gain feedback and confidence. Focus group and interview participants all spoke Khmer and were government volunteer CHWs working as part of the Village Health Support Group initiative identified above. They were selected by the Health Centre Chief based on their involvement with the malnutrition project and current level of activity as a CHW. Focus groups allowed CHWs to share ideas, compare experiences and comment on implementation of the project in Mondulkiri. The interviews provided an opportunity to express views from an individual perspective. CHWs associated with six health centres in Kratie were selected in collaboration with the Operational District Directors and Health Centre Chiefs based on their cultural (Muslim and Khmer communities), geographic (rural and town) and environmental (surrounded by river or central land mass) differences. Participant information sheets and consent forms were issued verbally and in written format and signed before any research activities took place. As the focus groups were part of an evaluation study, ethics approval was not necessary; however, full permission was gained from the National Ethics Committee for Health Research at the Cambodian Ministry of Health for the interviews which were part of a PhD study. The results were initially analysed and coded separately; however, given the similarity of the findings and emerging themes, a decision was made to combine the results in this paper. The process and reporting of this study followed the qualitative checklist from the Critical Appraisal Skills Programme (CASP) [60].

### Analysis

As described above, the collection and analysis of data began in the focus groups where participants collectively summarised and agreed on key solutions to identified problems and recorded them on flip charts. Involving participants in the analysis process is common in descriptive qualitative approaches and is stated to add depth and quality to the analysis process [61]. In addition, data analysed in the focus groups, researcher notes and written answers to interviews were analysed by the UK-based researcher. Following familiarisation, preliminary categories based on annotated notes were developed as the conceptual building blocks from which to construct theoretical structures. Data was coded by transferring data sections to the appropriate theme or sub-theme and then comparing the emerged coding’s together and in relation to the entire data set [61]. The data was then re-contextualised in terms of the themes, developing sub-themes where necessary [61, 62]. A thematic table was developed in MS Word and used to synthesis the findings [7, 63].

## Results

Focus group discussions lasted approximately 3 h, and flip charts were used to capture summaries which were verified by the participants as accurate. In addition, summary notes of key discussions were taken during the workshops by research assistants and the UK researcher which were included in the analysis process.

Table 2 shows the characteristics of the participants in the focus group.

**Table 2:** Characteristics of focus group participants

Characteristics	Focus group 1	Focus group 2
Gender	Five females, one male	Five females, one male
Age	Range 25–52 years Average age 32 years	Range 24–44 years Average age 32 years
Ethnicity/religion	6 Khmer	6 Khmer
Time as a CHW	Range 2–10 years, average 7.8 years	All CHWs 1 year or less

Interviews were semi-structured and included exploration of the roles, challenges and experiences of being a CHW in Cambodia. The RAs interviewed ten CHWs in Khmer, which were translated into English during the interview to allow for timely responses by the UK researcher. The UK researcher recorded answers in written format during the process. Table 3 shows the characteristics of the interviewees.

**Table 3:** Characteristics of interview participants

Characteristic	10 community health workers
Gender	Eight females (6 Khmer and 2 Cham Muslims) Two males (Khmer)
Age	Age range 26–65 years Average age 47 years
Other roles	Village Chief, Deputy Village Chief, works for commune council, farmer and seller, carer for partner and mother
Time as a CHW	Range between 4 and 24 years Average time as CHW 13 years

### Themes

Three main themes are presented here: roles and responsibilities, challenges and solutions. The first theme, roles and responsibilities, explores the health topics and activities undertaken by CHWs and their perceived responsibilities to community members and health facilities. The second theme highlights the challenges raised by CHWs when trying to fulfil their duties and are presented under three sub-themes; systemic challenges, personal challenges and community challenges. The final theme presents solutions identified by CHWs to the challenges they proposed and presents a table of actions that would assist them in their roles of improving community health.

### CHW roles and responsibilities

CHWs identified a number of roles and responsibilities as part of their volunteer position. These include responsibility to analyse and identify health issues arising in the community and communicate these to health centres and to share information from health centres with the community. Increasing health-seeking behaviour by signposting community members to health centres when problems arise is also a key activity. One CHW explained,

My role includes giving information to the villagers as well as giving information to the health centre if there was a problem in the community. I instruct people to go to the health centre if they are sick, or for pregnancy check or for delivery. (Khmer, male, age 61, CHW for approximately 22 years)

Others reported encouraging community members to seek health advice, as one CHW explains,

…people come to my house when they are sick and tell me about their symptoms, sometimes I know and tell them to go to the health centre. If they have a cold they go to a private pharmacy. For malaria, vaccinations and pregnancy checks they go to the health centre. (Khmer, female, age 26, CHW for approximately 5 years)

The CHW role requires a number of skills including effective communication and analytical ability to identify health issues arising in the community. CHWs also displayed a good working knowledge of their communities by identifying groups who required additional support, for instance, those who had not attended health facilities before:

I deliver letters to the mothers to tell them to take their children for a vaccine. I have a book of four vouchers that are for introducing people that have never been to the health centre before… (Muslim, female, age 53, CHW for approximately 15 years)

CHWs identify community members who meet the criteria for a government ‘user-fee exception programme’ and register them for a ‘poor card’:

There are a lot of people who have poor cards in the village. Everyone who is eligible has a poor card. Because me and another villager makes sure that if people are away working at the time of the poor card selection process, we make sure their name is down. (Khmer, female, age 26, CHW for approximately 5 years)

Another CHW explained how she negotiates fee exceptions with health centres when poor families were unable to pay:

If the villager is poor and no poor card and they are sick, I organise them to get free health care with the HC. (Khmer, female, age 39, CHW for 4 years)

**Fig. 1:**
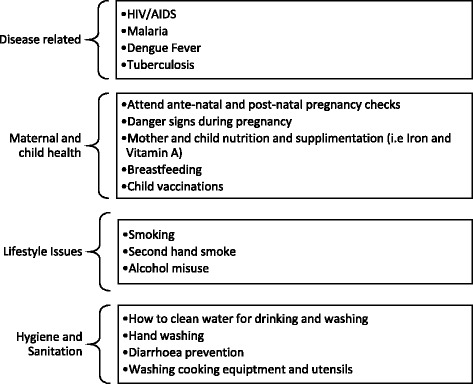
Health topics covered by CHWs

All CHWs deliver health promotion to community members on a number of health topics as shown in Fig. 1. One CHW explains some of the health issues she has covered in her practice:

Anything I learn from the workshops I then spread around the community, Malaria, TB, screening for pregnancy, new-born care, nutrition, vaccinations, vitamin A supplements and other health related information. I am a volunteer but I get paid $2 a day from the Provincial Health Department to find and refer infants for vaccinations. (Khmer, female, age 51, CHW for approximately 22 years)

Health promotion activities are delivered in several formats such as one to one or in a group setting. One CHW explained how her role as shop seller allows her to deliver health promotion in an opportunistic way:

I give education to villager and sometimes in a group and sometimes only 1 or 2 people. If people come to the shop to buy something I tell them the health info I have learnt. Last month I was giving advice about mosquito nets and to make sure there are no holes. I was giving education to people who go to cut the bamboo and told them they must take the mosquito net with them along with the hammock and to put it across the trees. Also, to wear clothes that has long sleeves to cover their body from mosquito. (Khmer, Female, age unknown, CHW for over 10 years)

When CHWs work with NGOs they are required to make plans and meet regularly with reports on activities:

We have a meeting with [named NGO] every two months where we are required to submit a report and to make plans for the following months. (Khmer, female, age unknown, CHW for over 10 years)

CHWs attend training at the national level in Phnom Penh, at the provincial level and with external agencies on a vast array of health topics (Fig. 1). Training formats include full-day workshops, short training sessions or meetings. Most training sessions are associated with small payments which varied depending on length of session and travel distance. NGOs are mentioned as the main training providers:

Two [NGO] organisations gave me the training and it was about pregnancy health and post-natal health. The training was three days long. I could only attend two days as the third day I gave birth to my son. After the training, I collect all the pregnant women and spread the messages I receive... (Khmer, female, age 26, CHW for approximately 5 years)

All CHWs from the focus groups had been trained on child malnutrition by an external agency in collaboration with the PHD, operational district (OD) and health centre. CHWs training experiences and attendance varied as there is no structured training programme.

### Challenges

CHWs from the focus groups identify systemic, personal and community challenges that impact on their ability to meet expectations.

### Systemic challenges

Systemic challenges include the lack of financial support, for example, when payment for training attendance is not sufficient to cover the loss of wages, childcare, travel and food costs. CHWs sometimes miss training sessions because they have other commitments; training is usually only delivered once. The lack of refresher training means they did not always remember what they had learned when it came to knowledge application over a period of time. Costs associated with programme mobilisation such as fuel for transport to community member’s homes or group activities came out of CHWs’ own pockets. They are not always supplied with the necessary equipment and promotional material (leaflets or posters) to conduct demonstrations or education required.

### Personal challenges

Most CHWs experience other demands on their time including work commitments and care responsibilities, for elders and children. It is normal practice to pay others to cover their work while they attend training, and sometimes the payment is not sufficient to cover this. Some CHWs also have civic duties such as being a Village Chief or Deputy. In addition, not all CHWs have access to transport and so rely on family to take them to training sessions and many commented that report writing is often a struggle and time-consuming.

### Community challenges

A lack of community interest and participation was reported as a significant challenge for CHWs. There was a number of reasons put forward for this including work commitments, a lack of respect of CHW knowledge and no interest in the topic area, compounded by a lack of incentives to attend health promotion sessions. Furthermore, the education level of community members is often low which challenges their ability to understand complex health information. Some community members were not familiar with linking poor health practices to ill health such as washing hands before eating or after ‘toilet’ use. Furthermore, CHWs feel they that their knowledge is often doubted.

I tell the women not to smoke in the house or the children get asthma bad and I tell them it is because of the smoke but they don’t believe it. (Muslim, female, age 34, CHW for 15 years)

Traditional healers are still recognised by rural communities as a popular source of health knowledge, which can be in conflict to information provided by the CHWs. Many CHWs feel disempowered by the lack of a professional title and uniform, which could help validate their role in the community. Table 4 summarises the community challenges identified by CHWs during the focus groups.

**Table 4:** Community engagement challenges

Challenges to engaging the community in health promotion sessions	
1. Community members are busy with work and home duties and so do not have time to engage in group education 2. Community members do not believe health promotion messages or do not respect CHW knowledge 3. Community members are not interested in health topics 4. No incentives for community members to attend health promotion sessions 5. Difficulty in understanding the link between poor health practices and ill health	

CHWs feel it is a struggle to achieve behaviour change in community members following health promotion sessions. CHWs explained their thoughts on why attendees would not cook nutritional porridge to improve child nutrition following sessions teaching them how to make it. These were disbelief in the messages being promoted, family working practices leave little time for planning and cooking, insufficient money to afford nutritious food, a lack of capacity to plan and sustain a small vegetable garden to improve access to nutritious foods and engrained habits such as traditional feeding practices.

### Solutions

The CHWs suggest improvements and possible solutions to the challenges presented which are summarised in Table 5. CHWs believe that behaviour change can only be achieved with repeated health promotion sessions. One-off sessions or group demonstrations are not enough to achieve behaviour change and break old habits. Also expressed is the need to show villagers the consequences of not changing health practices; for instance, simply providing demonstrations of cooking nutritional porridge for children is not sufficient and caregivers must understand the link between the foods and improved child health. The idea of ‘model families’ or ‘real-life people’ demonstrating how they made positive changes and how it affected them is considered by several CHWs as a good idea.

**Table 5:** Suggested improvements for health programmes by CHWs

Challenge	CHW-suggested improvements
Low community attendance at health promotion session	Spend time before health promotion sessions informing community members of the importance of the session and the link between ill health and the public health issue
	Offer incentives to attract people to group sessions (bar of soap, small snack)
	Encourage respected members of the community to promote education sessions, i.e. village chiefs and deputies, monks, school staff
	Have repeated health promotion sessions to enable more community members to attend and to help engrain new messages for those with low education levels
	Keep all health promotion language simple and consistent
	Provide CHWs with a uniform and fully integrate into the health system to increase respect from the community
Behaviour change outcomes	Work in partnership with other providers, for instance, NGOs working on sustainable crop programmes can help community members to grow their own produce to reduce malnutrition in under-five
	Follow-up group health promotion sessions with individual home visits and observations
	Reinforce messages using TV/radio and other media
	Show real-life examples of local people who have made the change and what the benefits are for them
CHW training	Offer training sessions multiple times to increase CHW attendance and offer refresher training regularly
	Ensure payment to attend training days is sufficient for CHWs to attend
Resources and ongoing support	Provide full resources to mobilise programmes including equipment, a travel mode, fuel fund and media resources such as leaflets and posters
	Local government to raise the importance of health issues and demonstrate their support to local communities
	Raise health issues at commune meetings to generate interest and support
	Local health officials to recognise CHWs and provide them with an identity
Difficulty in reporting	Include verbal reporting mechanisms to decrease the amount of time spent by CHWs on written reports

It is felt that if CHWs had T-shirts with a respected logo, perhaps from the Ministry of Health, they would have more of an identity with the health system and gain more respect from the villagers. It is also suggested that if the village chief and local government, either the PHD or OD, fully support and promote the demonstrations, more villagers would attend.

CHWs identify a need for more coordination between NGOs trying to achieve similar health improvements. For instance, a local NGO working on sustainable farming and crop growing would improve access to nutritious foods for families, which in turn would improve the nutritional status of children. The focus group sessions recorded the solutions to challenges in Table 5.

## Discussion

The CHWs in this study portrayed a range of training and interventions to address public health issues faced by communities and explained how they use that knowledge to deliver health promotion in a variety of ways to improve health practices. However, some of the challenges faced by CHWs in Cambodia hinder their ability to optimise their performance. The following discussion explores the challenges and solutions identified by CHWs in relation to literature, highlighting the importance of government ownership, identity, adequate training and resources. Finally, the need for CHWs to be consulted on programme planning and delivery is discussed.

### Government ownership and CHW identity

The challenges identified by CHWs such as insufficient remuneration to attend training and deliver activities, irregular one-off training sessions without opportunities to refresh knowledge, inadequate resources and travel difficulties have been reported to demotivate and reduce CHW performance in other LMICs [14, 15, 38, 64]. In Cambodia, as is the case in countries who are largely dependent on international aid, the structure, training, delivery and support of CHWs is mainly delivered by external agencies such as NGOs [16, 37, 50, 65]. A consequence of this is that CHW programmes have ill-defined ownership and lack government accountability, leadership and management WHO; [4]. Active government ownership and leadership with good programme management is a key factor in successful CHW programmes that is often neglected in research and practice [3, 4, 66, 67]. Furthermore, as evidenced here, the association of CHWs as extensions of NGO programmes further distances them from the health system [65]. CHWs have been reported to feel empowered when they are associated with the health system and viewed as a credible source of information [14, 64, 68]. Here, they express a desire to have more involvement from the local government such as endorsing their health promotion sessions, supplying them with a uniform and ID and delivering media campaigns that would re-enforce the messages they are trying to promote.

To address credibility and alignment with the health system, several methods have been suggested in the literature. These include media campaigns highlighting the roles and responsibilities of CHWs, which were found to successfully build their image as a credible source of health information [69]. Mass media health campaigns about specific health issues being delivered at the same time as CHWs also emphasised their knowledge in the eyes of the community [69]. Clear government ownership of CHW programme delivery that identifies CHWs as a valuable part of the health workforce through adequate support, such as that requested by CHWs here, is likely to facilitate a more coordinated approach and structure of training, remuneration and resources.

### CHW training and resources

The success of CHW programmes is dependent on common design factors such as initial and regular training, supportive supervision and career development opportunities [6, 14, 38, 64, 70–72]. Successful CHW programmes include ‘Lady Health Workers’ in Pakistan who undergo 15 months of training and 3 months full time followed by 12 months of in-service training [73]. They also receive a small allowance, have dedicated supervisors and are attached to a government health facility, from which they receive training [73]. Similarly, in Iran and Thailand, training includes theory and practical classes covering health promotion, disease prevention, communication skills and social determinants of health [57, 74]. Initial core training packages for CHWs are recommended along with accreditation and financial incentives [5, 7, 57, 75]. In Cambodia, there is not an accreditation system, initial training package or regular structured ongoing training and remuneration [17]. Therefore, basic crosscutting public health skills required for their role such as health promotion, behaviour change communication and public engagement techniques are not delivered in a structured manner.

CHWs highlight their frustration that community members did not change their behaviour following health promotion sessions on how to cook nutritional porridge for children. Research has highlighted that programmes developed to improve infant feeding practices such as the one delivered here, often fail to consider theories of behaviour change [76]. If CHWs had received training on behaviour change techniques, they might have been able to better address the issue of community adherence [69]. International agencies have recognised the need to equip CHWs in Cambodia with behaviour change communication skills. For example, in 2005, a 6-year, nationwide Behaviour Change Communication (BCC) skills development programme for health centre staff and CHWs was delivered in Cambodia [45]. The project trained local government staff to deliver BCC training using tools and resources developed with international partners. However, high staff turnover, insufficient remuneration, low motivation, a lack of government ownership and sustainable funding hindered the project’s success. Future sustainability was jeopardised by the growing verticalisation of health programmes in Cambodia, where strategic commitment to deliver such crosscutting skills was dwindling [45]. The evaluation described doubts that sustainability was possible. Initial training followed by regular ongoing refresher training is said to be essential to the performance and quality of service provided by CHWs [4, 14, 38, 66]. A core training package including techniques for community engagement, health promotion and counselling which is government planned, financed and owned is recommended [5].

CHWs here express a need for adequate resources to deliver ongoing group health promotion sessions to expose communities to messages on more than one occasion. This includes a travel mode such as a bike or fuel for a motorbike, leaflets and posters and in some cases cooking equipment for demonstrations. However, due to the funding streams of many initiatives, group education and resources were planned for the short term and lacked long-term sustainability. Not only are the CHWs restricted to what they can deliver because of this but research has shown that a lack of resources also risks reducing community respect, a problem highlighted by the CHWs here [7, 14]. There is ongoing discourse on allowances for CHWs including a regular paid wage and adequate tools to do their job [15, 77]. CHWs in this research did not speak about a regular wage but of needing fuel, equipment and sufficient payment for training when they were required to leave work or family for extended periods of time. A lack of resources is considered a major contributor to the failure of CHW programmes globally [15, 38, 77].

### CHW capacity to develop practical solutions

CHW programmes are considered more successful when they are involved in planning, implementing and monitoring processes through shared ownership and resources [66, 78, 79]. Scott and Shanker state that CHWs could be powerful change agents when affiliated with health systems able to adequately support their work and respond to their insights [9]. Current systems of CHW management are not optimising their ability to contribute to health improvement [80]. The voices of CHWs are often silent or are not considered as acceptable evidence to shape policy and procedure [19, 71]. CHWs here demonstrate their capacity to identify possible links between programmes and agencies to achieve similar outcomes. This was shown in their reference to joining a sustainable farming and crop growing NGO with the under-five malnutrition programme to increase access to nutritious foods.

The insights shown in the solution table (Table 5) provide evidence that CHWs, when facilitated to consider improvements to programme delivery, can provide practical realistic solutions. Although some of the solutions identified would have associated costs, there are potential savings from implementing a more effective programme. A cost analysis of all interventions suggested by CHWs is recommended to ascertain feasibility and practicality. However, opportunities for CHWs to suggest solutions are not always presented by organisations, or if they are, it is part of an evaluation and not an ongoing process. Once insights are established, it is important that suggestions provided by CHWs are supported. If CHWs are to contribute towards the improvement of health in rural communities, they must feel empowered to be the voice of the community, be listened to and respected by those that have power and resources [68]. Policy makers would benefit from listening and applying the suggestions voiced by CHWs [54].

### Limitations

This study only presents the challenges and solutions as identified by CHWs and does not include that of other stakeholders. Also, the sample sizes are low and from only two provinces; other CHWs based in other provinces might have differing views. It is also recognised that the CHWs were chosen by health centre chiefs, and this could create bias. However, this was done to ensure that participants were active as CHWs and were involved in relevant projects which would maximise communication of current experiences. Finally, as this was a cross-language study, some meaning might have been compromised in the translation process.

## Conclusion

CHWs in Cambodia are a valuable workforce and so clearly have a potential role in improving community health. However, without a more structured delivery system that allows them to be part of the planning and development process, their performance is compromised [65]. The lessons learned here may be applied more widely to assist health programmes that involve CHWs in Cambodia and other countries. Stronger CHW identity and a more structured induction training and support programme led by local government bodies that includes basic crosscutting skills such as communication and behaviour change techniques would likely improve practice [5, 69, 81]. CHWs require adequate resources and tools to do their jobs as part of a long-term ongoing strategy. This will allow them to deliver health promotion sessions on a regular basis rather than as a one off to meet the needs of short-term externally planned health programmes [10]. Further research is required to understand how to maximise CHW input so that they may generate insights to inform planning and delivery of health programmes, thus optimising their capacity to improve health in Cambodia.

## Abbreviations

CHW: Community health worker
INGO: International non-government organisation
LMIC: Low andMiddle-income country
NCD: Non-communicable disease
NGO: Non-government organisation
OD: Operational district
PHD: Provincial health department
RA: Research assistant
TB: Tuberculosis
VHSG: Village Health Support Group
